# Dysfunction of the adhesion G protein-coupled receptor latrophilin 1 (ADGRL1/LPHN1) increases the risk of obesity

**DOI:** 10.1038/s41392-024-01810-7

**Published:** 2024-04-26

**Authors:** André Nguyen Dietzsch, Hadi Al-Hasani, Joachim Altschmied, Katharina Bottermann, Jana Brendler, Judith Haendeler, Susanne Horn, Isabell Kaczmarek, Antje Körner, Kerstin Krause, Kathrin Landgraf, Diana Le Duc, Laura Lehmann, Stefan Lehr, Stephanie Pick, Albert Ricken, Rene Schnorr, Angela Schulz, Martina Strnadová, Akhil Velluva, Heba Zabri, Torsten Schöneberg, Doreen Thor, Simone Prömel

**Affiliations:** 1 Rudolf Schönheimer Institute of Biochemistry, Medical Faculty, Leipzig University, Leipzig, Germany; 2 Institute for Clinical Biochemistry and Pathobiochemistry, Medical Faculty, German Diabetes Center, Leibniz Center for Diabetes Research at Heinrich Heine University Düsseldorf, Düsseldorf, Germany; 3 German Center for Diabetes Research (DZD e.V.), Munich-Neuherberg, Germany; 4 Cardiovascular Degeneration, Clinical Chemistry and Laboratory Diagnostics, Medical Faculty, University Hospital and Heinrich Heine University Düsseldorf, Düsseldorf, Germany; 5 Cardiovascular Research Institute (CARID), Medical Faculty, University Hospital and Heinrich Heine University Düsseldorf, Düsseldorf, Germany; 6 Institute of Pharmacology, Medical Faculty, University Hospital and Heinrich Heine University Düsseldorf, Düsseldorf, Germany; 7 Institute of Anatomy, Medical Faculty, Leipzig University, Leipzig, Germany; 8 Center for Pediatric Research, Hospital for Children and Adolescents, Medical Faculty, Leipzig University, Leipzig, Germany; 9 Helmholtz Institute for Metabolic, Obesity and Vascular Research (HI-MAG) of the Helmholtz Zentrum München at the University of Leipzig and University Hospital Leipzig, Leipzig, Germany; 10 Department of Endocrinology, Nephrology, Rheumatology, Leipzig University Medical Center, Leipzig, Germany; 11 Institute of Human Genetics, Leipzig University Medical Center, Leipzig, Germany; 12 Institute of Cell Biology, Department of Biology, Heinrich Heine University Düsseldorf, Düsseldorf, Germany; 13 School of Medicine, University of Global Health Equity, Kigali, Rwanda

**Keywords:** Endocrine system and metabolic diseases, Physiology

## Abstract

Obesity is one of the diseases with severe health consequences and rapidly increasing worldwide prevalence. Understanding the complex network of food intake and energy balance regulation is an essential prerequisite for pharmacological intervention with obesity. G protein-coupled receptors (GPCRs) are among the main modulators of metabolism and energy balance. They, for instance, regulate appetite and satiety in certain hypothalamic neurons, as well as glucose and lipid metabolism and hormone secretion from adipocytes. Mutations in some GPCRs, such as the melanocortin receptor type 4 (MC4R), have been associated with early-onset obesity. Here, we identified the adhesion GPCR latrophilin 1 (ADGRL1/LPHN1) as a member of the regulating network governing food intake and the maintenance of energy balance. Deficiency of the highly conserved receptor in mice results in increased food consumption and severe obesity, accompanied by dysregulation of glucose homeostasis. Consistently, we identified a partially inactivating mutation in human ADGRL1/LPHN1 in a patient suffering from obesity. Therefore, we propose that LPHN1 dysfunction is a risk factor for obesity development.

## Introduction

The worldwide prevalence of obesity is continuously rising, with more people currently affected by overweight than underweight.^1^ It is well established that obesity contributes to major health problems such as type 2 diabetes and cardiovascular diseases, ultimately leading to increased morbidity and mortality.^2^ Obesity has multifactorial causes, and its onset and severity are influenced by environmental, social, and lifestyle factors, as well as genetic predisposition.^3^ Therefore, the identification of altered components and pathways that increase the risk of obesity is pivotal for designing patient-specific prevention and treatment strategies.

Proper regulation of food intake is central for maintaining energy homeostasis and, thereby, to prevent obesity. This process involves a variety of neuropeptides and hormones. Central regulation of energy homeostasis occurs in the hypothalamus, with the arcuate nucleus considered the master regulator of hunger and satiety.^4–6^ It contains two sets of neurons that play key roles in synthesizing and releasing peptides that regulate appetite: the POMC- and the AGRP/NPY neurons. POMC neurons secrete proopiomelanocortin (POMC)-derived peptides (e.g., the α-melanocyte-stimulating hormone (α-MSH)), while AGRP/NPY neurons release agouti-related protein (AGRP) and neuropeptide Y (NPY).^7^ However, these neuropeptides also regulate the activity of other neurons and the secretion of additional hormones. As such, anorexigenic effects are mediated by cocaine- and amphetamine-regulated transcript (CART) peptides, corticotropin-releasing hormone, oxytocin,^8^ and neurotensin,^9^ while melanin-concentrating hormone, orexins, and galanin promote food intake.^10^ All these processes are modulated by numerous receptor-mediated signaling cascades, with G protein-coupled receptors (GPCRs) and their signaling pathways being among the most prominent ones.^11–13^ Several GPCRs play functional roles in regulating energy homeostasis by modulating neuronal activity, such as the ghrelin receptor (GHSR),^14^ NPY receptor type 2 (NPY2R),^15^ serotonin receptor type 2 (5-HT2),^16,17^ GPR30,^18^ and the melanocortin receptor type 3 (MC3R).^19^

In addition to these centrally controlled mechanisms, metabolic processes in peripheral organs also contribute to the energy balance. For example, several hormones secreted from adipose tissue, such as leptin and resistin, modulate food intake and energy expenditure.^20,21^ Moreover, increased concentrations of free fatty acids regulate the activity of hypothalamic neurons via the free fatty acid receptors FFAR1 and FFAR4.^22^ Furthermore, the function of adipocytes is regulated by a plethora of GPCRs, with β-adrenergic receptors being the most extensively studied. Activation of this receptor family leads to an increase in intracellular cAMP, subsequently reducing insulin-stimulated glucose uptake and modulating hormone secretion as well as enhancing lipolysis and thermogenesis in white and brown adipocytes.^23^ Similar effects have been described for the adenosine receptor A2a,^24,25^ whereas the Gi-coupling receptor A1 has the opposite effect on lipolysis.^24^ Activation of G_q_-coupled pathways stimulates insulin-dependent glucose uptake and simultaneously inhibits lipolysis, as shown for FFAR2 and FFAR4.^26,27^

These roles of GPCRs highlight their importance in regulating food intake and energy balance through both central and peripheral mechanisms. As many GPCRs are valuable pharmacological targets, efforts are constantly being made to identify novel receptors involved in these processes. Consequently, expression and functional analyses are used to identify and evaluate orphan GPCRs in this context.^28–30^

In the present study, we have expanded this search to include members of a so far grossly neglected class of GPCRs, the adhesion GPCRs (aGPCRs). These receptors are structurally and functionally unique, with extraordinarily large extracellular N termini enabling various molecular functions such as cell adhesion and signal transduction (summarized in ref. ^31^). These functions are essential in numerous biological contexts and, when dysfunctional, can lead/contribute to severe diseases such as neurological disorders^32–34^ or cancer (summarized in^35^). Recently, aGPCRs have been shown to play roles in metabolism (summarized in ref. ^36^). Interestingly, we found that the aGPCR latrophilin 1 (ADGRL1/LPHN1) is involved in controlling energy balance and food intake. Although this receptor is present in adipocytes^37^ and many neurons,^38,39^ where it is essential for axon growth cone formation^40^ and neuronal function (summarized in ref. ^41^), the details of its involvement in the regulation of energy balance or food intake have not been previously described. Here, we demonstrate that the lack of *Lphn1* leads to severe obesity in mice, likely caused by overeating. Consistent with these results, we identified partial loss-of-function variants of the human *LPHN1*, one of which occurred in a child with obesity. Our data highlight ADGRL1/LPHN1 as a novel regulator of food intake and energy balance, and its dysfunction may contribute to the onset or progression of obesity.

## Results

### Mice lacking LPHN1 develop obesity

**Fig. 1:**
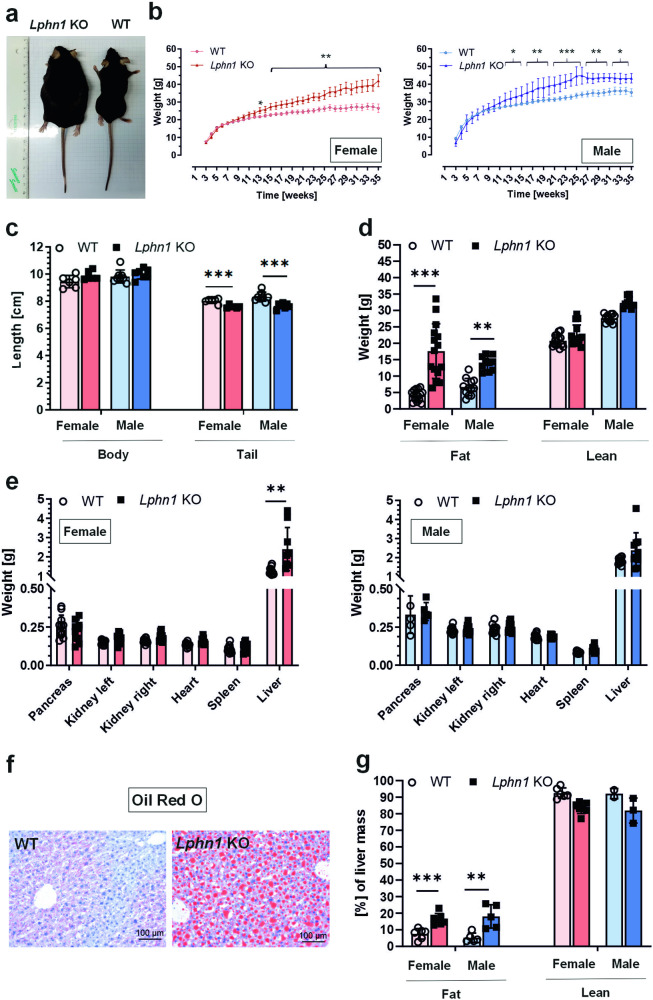
*Lphn1*-deficient mice display an increased weight and fat accumulation. **a** *Lphn1* knockout (KO) mice are bigger than wild-type (WT) littermates. Representative images of 30-week-old female mice on a cm scale. **b** *Lphn1* knockout mice gain more weight over time than wild-type littermates. Mice were weighed weekly. Weight differences become visible after 12 weeks in females as well as males and increase with age. Data are given as means ± SD; *n* ≥ 8; *p < 0.05; **p < 0.01; ***p < 0.001. **c** The body length of *Lphn1* knockout mice is indifferent from the one of wild-type littermates, but tails are shorter. Body and tail lengths were measured in mice 30–34 weeks of age. Given are means ± SD; *n* = 5-6; ***p < 0.001. **d** *Lphn1* knockout mice have more fat, but similar lean body mass. Body composition of 30–34-week-old mice was determined using EchoMRI. Given are means ± SD; *n* ≥ 10; **p < 0.01; ***p < 0.001. **e** Organ mass of *Lphn1* knockout and wild-type mice are similar. Only the livers are significantly heavier in *Lphn1* knockout animals (30–34-weeks of age). Data are means ± SD; *n* ≥ 8; **p < 0.01. **f** Representative Oil red O staining of liver sections of 30–34-week-old male mice used to directly visualize the stored triglycerides in liver cells (red). The staining reveals high numbers of large lipid droplets within *Lphn1* knockout livers. **g** Livers of *Lphn1* knockout mice contain more fat than wild-type livers. Liver fat and lean body mass of 30–34-week-old animals was determined by EchoMRI. Data are given as means ± SD; *n* ≥ 5; **p < 0.01; ***p < 0.001

Mice constitutively lacking *Lphn1*^42^ become severely overweight with age. While shortly after weaning, no weight difference was detected in *Lphn1*-deficient mice compared to wild-type animals, from the age of 5–9 weeks onwards, they gained approximately 1.7-fold more weight than wild-type littermates (Fig. 1a, b). This effect was observed in both sexes but appeared to be more pronounced in females.

To identify the cause of the increased weight, we first measured the animals’ body length, which was unaltered in the absence of LPHN1 (Fig. 1c) suggesting no effect on overall growth. Interestingly, *Lphn1* knockout mice had shorter tails than wild-type controls (Fig. 1c), a phenomenon also reported in mice with overweight caused by a loss-of-function mutation in the leptin receptor.^43^ Next, we investigated whether the increased weight was due to elevated fat mass and determined the fat-lean body mass composition of 30–34-week-old mice using EchoMRI. These analyses revealed that *Lphn1*-deficient animals displayed a significant increase in fat body mass, but not in lean body mass (Fig. 1d).

Consistent with these results, the organ weights of *Lphn1* knockout mice were indistinguishable from those of wild-type controls (Fig. 1e), suggesting that the increased body weight was mainly caused by fat accumulation. Only the livers of *Lphn1* knockout mice were significantly heavier. As the liver is one of the most essential storage organs, this greater liver weight might also result from increased fat storage. Oil red O staining showed severe morphological differences between knockout and wild-type livers confirming the accumulation of lipids by the appearance of droplets (Fig. 1f). These findings were further substantiated by liver composition analyses with EchoMRI, which showed that *Lphn1* knockout livers displayed a higher fat content (Fig. 1g).

In conclusion, the absence of LPHN1 leads to mice becoming heavily overweight over time by accumulating excess fat.

### LPHN1 modulates food intake and energy balance

**Fig. 2:**
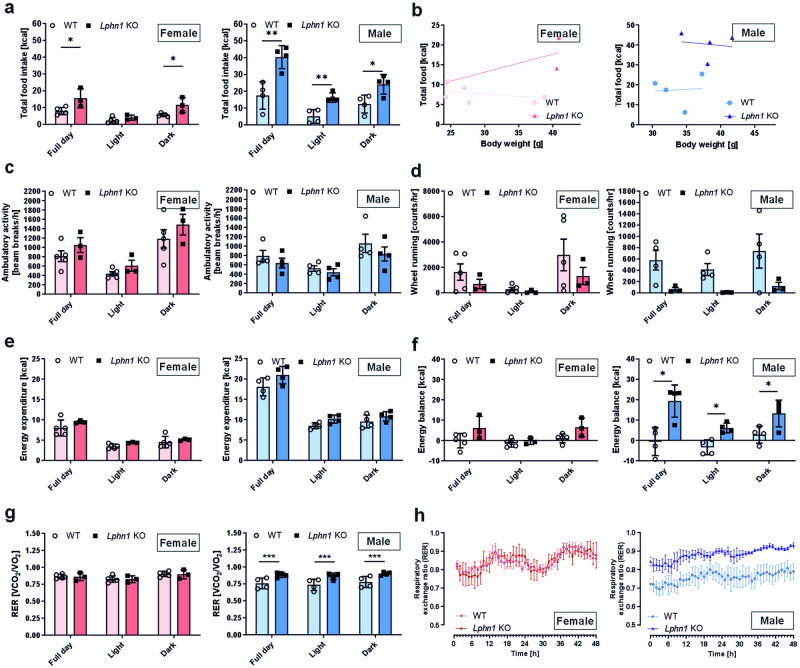
Loss of *Lphn1* increases food intake in male and female mice and alters energy metabolism in male mice. **a** Food intake during both the light and the dark phase is increased in *Lphn1* knockout (KO) mice compared to wild-type (WT) littermates in male and female animals. **b** Analysis of food intake using body mass as a covariate shows that loss of LPHN1 rather than increased body weight is causative (ANCOVA, female: p = 0.09, male: p = 0.03). **c** 30-34-week-old *Lphn1* male and female knockout mice show similar levels of movement activity than wild-type (WT) littermates independently of light or dark phase. **d** Wheel running activity shows a trend towards reduction in *Lphn1* knockout animals. **e** Energy expenditure does not show significant differences in *Lphn1* knockout mice compared to wild-type littermates. **f** The overall energy balance (food intake minus energy expenditure) is significantly increased in male *Lphn1* knockout mice during both the light and dark phases. **g**, **h** The respiratory exchange rate in male *Lphn1* KO mice is significantly higher than in WT controls during light and dark phase whereas no change is obvious in female animals. Data in are given as means ± SD of three to five 30–34-week-old animals; *p < 0.05; **p < 0.01; ***p < 0.001

As weight gain often results from imbalanced energy homeostasis and/or increased calorie uptake, we hypothesized that LPHN1 plays a role in controlling one of these underlying processes. To test this hypothesis, we investigated the feeding behavior and energy metabolism of *Lphn1* knockout mice using indirect calorimetry in metabolic cages. In the absence of the receptor, mice on a standard diet consumed significantly more food than wild-type littermates, as indicated by food intake (Fig. 2a). This increase was more pronounced in males than females. In knockout male mice, food intake was significantly higher compared to WT controls (p = 0.0293), as analyzed by ANCOVA using body mass as covariate (Fig. 2b). Although they displayed a similar level of ambulatory activity (Fig. 2c), there was a trend towards reduced wheel running activity of *Lphn1* knockout mice in both males and females (Fig. 2d). *Lphn1*-deficient mice showed a slight increase in energy expenditure, although it was not statistically significant (Fig. 2e). These alterations led to a permanently increased energy balance (food intake minus energy expenditure) in *Lphn1* knockout mice (Fig. 2f) indicating that LPHN1 is involved in the regulation of food intake. Additionally, in the absence of LPHN1, male mice showed a significant increase in the respiratory exchange rate (RER) compared to wild-type controls. This difference was observed during both the light and dark phases (Fig. 2g, h). Consistently, whole-body fat oxidation (FAO) rates were reduced (supplementary Fig. S1a), while whole-body carbohydrate oxidation (CHO) was significantly increased in *Lphn1* knockout mice (supplementary Fig. S1b). This change was absent in female animals (supplementary Fig. S1a, b).

Our data indicate that LPHN1 plays a role in the control of food intake independent of sex. Furthermore, it is involved in regulating substrate utilization preference in male animals.

### Lack of LPHN1 does not affect the expression of different hypothalamic neuropeptides

**Fig. 3:**
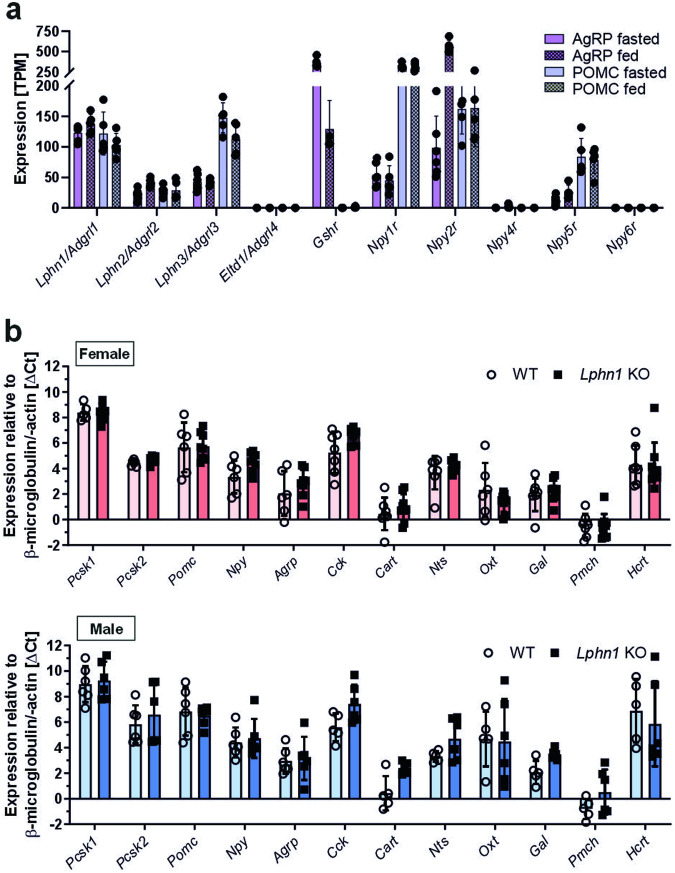
LPHN1 is present on hypothalamic neurons that regulate feeding behavior. **a** Meta-analysis of RNA-seq data from^45^ revealed expression of all three latrophilin homologs in mouse AGRP- and POMC neurons. The mRNA levels of *Lphn1* and *Lphn3* are comparable to those of some GPCRs (*Gshr* and *Npy1R*–*Npy6R*) known to be involved in the regulation of food intake. *Eltd1*, the fourth member of this aGPCR group, is not expressed. Given are mean transcript per million mapped reads (TPM) values ± SD of five individuals. **b** The expression levels of neuronal components involved in the regulation of food intake are not altered in obese *Lphn1*-deficient mice. The hypothalamus of 30–34-week-old mice were subjected to qPCR analysis using gene-specific primers for prohormone convertase 1 (gene *Pcsk1*), prohormone convertase 2 (gene *Pcsk2*), the orexigenic neuropeptides NPY, AGRP, galanin (gene *Gal*), pro melanin concentrating hormone (gene *Pmch*), and hypocretin (gene *Hcrt*) as well as the anorexigenic neuropeptides POMC, Cart, neurotensin (gene *Nts*), cholecystokinin (gene *Cck*), and oxytocin (gene *Oxt*). Data are given as means ± SD; *n* ≥ 3 normalized to β-microglobulin/-actin (20.60 ± 2.33)

There are several causes conceivable that might contribute to the alterations observed in food consumption of *Lphn1*-deficient mice. One possible explanation is dysregulation of satiety and hunger in brain neurons, similar to what is observed for the MC4 receptor (MC4R) or the NPY receptor system. Numerous studies have shown that latrophilins are expressed in diverse areas of both the mouse (summarized in ref. ^44^) and human (Human Protein Atlas, proteinatlas.org) brains. All three receptor subtypes (ADGRL1-3/LPHN1-3) are also located in the hypothalamus, and existing transcriptome data^36,45^ showed their presence in both mouse AGRP- and in POMC neurons. *Adgrl4/Eltd1*, the fourth receptor in the ADRGL group of aGPCRs (Fig. 3a), was not detected. *Lphn1* showed the highest expression in both neuron types regardless of the feeding status. As AGRP- and POMC neurons are the two major types of neurons in the hypothalamus involved in the regulation of food intake, it is conceivable that LPHN1 plays a role in this process. Therefore, we investigated whether components involved in this regulation were differentially expressed in *Lphn1*-deficient mice. Initially, we focused on the prohormone convertases 1 and 2 (PC1/PC2), which process prohormones such as POMC into biologically active peptide hormones, as well as the three major neuronal players POMC, AGRP and NPY. However, we did not observe any significant changes in the expression levels of these food intake regulators (Fig. 3b). Since the regulation of energy homeostasis is not limited to these neuropeptides, we further analyzed expression of anorexigenic (cholecystokinin (*Cck*), cocaine- and amphetamine-regulated transcript (*Cart*), neurotensin (*Nts*), and oxytocin (*Oxt*)) as well as orexigenic (galanine (*Gal*), melanin-concentrating hormone (*Pmch*), and orexin (*Hcrt*)) hypothalamic neuropeptides. Again, we did not observe significant differences, indicating that LPHN1 might be involved in the control of food intake via currently unknown processes.

### *Lphn1* deficiency results in decreased glucose tolerance and insulin sensitivity

**Fig. 4:**
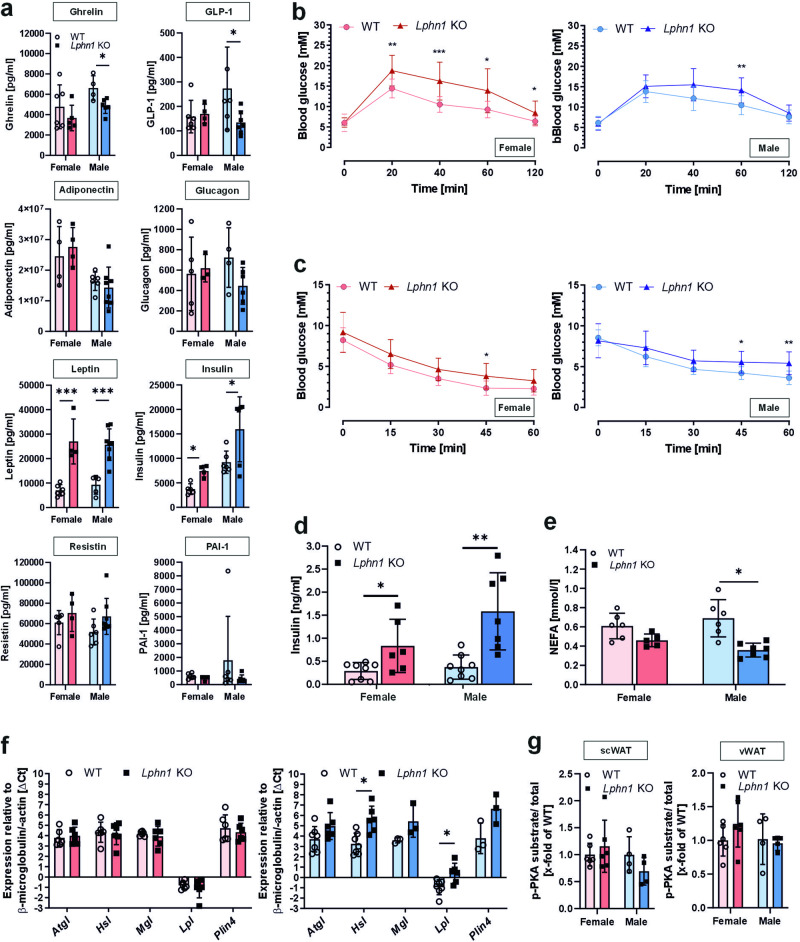
*Lphn1* knockout mice show changes in hormone levels controlling metabolism and an altered glucose and insulin tolerance as well as lipolysis. **a** Several metabolism-related parameters are altered in *Lphn1* knockout (KO) compared to wild-type (WT) littermates. Parameters were determined from blood serum samples of 30–34-week-old male mice. Serum levels are given as means ± SD; *n* = 5–8; *p < 0.05; ***p < 0.001. **b** *Lphn1* knockout male and female mice have a reduced glucose tolerance. Glucose tolerance tests were performed by intraperitoneal injection of 1 mg glucose/g body weight in mice of 30–34 weeks of age. Blood glucose levels are given as means ± SD; *n* ≥ 13; *p < 0.05; **p < 0.01; ***p < 0.001. **c** In the absence of LPHN1, mice show higher glucose levels after insulin administration. Blood glucose was measured after intraperitoneal injection of 1 mU insulin/g body weight. Data are given as means ± SD; *n* ≥ 8; *p < 0.05; **p < 0.01. **d** Plasma insulin contents are higher in overnight fasted *Lphn1* knockout mice than in wild-type littermates. Levels are given as means ± SD; *n* ≥ 7; *p < 0.05; **p < 0.01. **e** Serum NEFA levels in obese *Lphn1* knockout mice are reduced. Data are given as means ± SD; *n* ≥ 10. **f** Adipose tissue of 30–34-week-old mice was subjected to qPCR analysis using gene-specific primers. Expression levels of the lipases adipose triglyceride lipase (*Atgl*), hormone sensitive lipase (*Hsl*), monoacylglycerol lipase (*Mgl*), and lipoprotein lipase (*Lpl*) as well as perilipin 4 (*Plin4*) were assessed in visceral adipose tissue of female and male mice. Here, we observed no expression changes in females whereas expression is significantly altered for *Hsl* and *Lpl* in male *Lphn1*-deficient mice. Data are given as means ± SEM; *n* ≥ 3 normalized to β-microglobulin/-actin (18.62 ± 0.39). Note that high ΔCt values correspond to low expression and vice versa. **g** Phosphorylation levels of different PKA substrates in visceral (vWAT) and subcutaneous (scWAT) white adipose tissue in *Lphn1* KO mice, as assessed by a broad phospho-PKA-substrates antibody. In scWAT samples of male mice, a trend towards a reduced fraction of phosphorylated PKA substrates is visible. Quantification of Western blot images from Supplementary Fig. S2

Altered food intake, energy balance, and subsequent obesity is accompanied by changes in the levels of hormones and proteins controlling the energy status secreted by adipose tissue and other organs. For example, in obese individuals leptin, insulin, and resistin levels are increased,^46,47^ while adiponectin, ghrelin, and GLP-1 amounts are usually reduced.^48,49^ To address the question whether the absence of LPHN1 and the concomitant obesity in mice have a similar effect on these hormones, we measured blood serum levels of different adipokines (Fig. 4a). While ghrelin and GLP-1 were significantly reduced in male *Lphn1* knockout mice, a trend towards a reduction was also visible for adiponectin and glucagon. Similarly, significantly elevated serum levels of leptin and insulin were detected in male as well as female animals. We did not observe any significant changes in the regulation of plasminogen activator inhibitor-1 (PAI-1), an adipogenic factor known to be upregulated in obesity (summarized in^50^).

Changes in these parameters, especially in adiponectin, insulin, and leptin, can cause alterations in metabolic processes such as glucose uptake and metabolism. We then investigated the glucose tolerance of obese Lphn1 knockout mice. To exclude any effect on enteral glucose uptake, glucose was administered intraperitoneally. Following glucose injection, *Lphn1*-deficient mice showed significantly higher blood glucose levels than wild-type littermates indicating glucose intolerance in knockout mice (Fig. 4b). This effect was more pronounced in females than in males.

Higher glucose levels in individuals with obesity are often caused by decreased insulin sensitivity. To assess whether decreased insulin sensitivity was present in *Lphn1* knockout mice, we performed insulin tolerance tests. After insulin injection, blood glucose levels remained significantly higher in knockout mice when compared with wild-type mice, indicating insulin resistance in knockout mice. (Fig. 4c).

Interestingly, while our data did not indicate increased fasting glucose levels (Fig. 4b, c), we did observe increased basal insulin levels in *Lphn1* knockout mice (Fig. 4d).

These findings demonstrate that *Lphn1* deficiency leads to altered serum levels of adipokines and hormones regulating metabolism, a reduced glucose tolerance, and a decreased insulin sensitivity. All these changes are most likely caused by the obese phenotype of the *Lphn1* knockout mice.

### The absence of *Lphn1* causes mild alterations in lipolysis in male mice

As its expression in adipose tissue has been previously shown,^37^ it is also reasonable to assume that LPHN1 affects food intake and energy balance via different routes, such as the regulation of lipolysis. Therefore, we assessed lipolytic activity by determining serum levels of non-esterified fatty acids (NEFA). Indeed, male *Lphn1* knockout mice exhibited a one-third reduction in NEFA levels compared to wild-type controls (Fig. 4e). We further measured transcript levels of the main lipases releasing fatty acids in visceral adipose tissue. Interestingly, we found no changes in female mice, while in male animals, we observed a significant increase in ΔCt values of hormone sensitive lipase (*Hsl*) and lipoprotein lipase (*Lpl*) in the absence of LPHN1 (Fig. 4f) suggesting that the receptor might affect lipolysis. As the activity of many of these lipases, and thus of lipolysis, is controlled mainly by phosphorylation of the protein kinase A (PKA), we next evaluated whether this phosphorylation is altered in the absence of LPHN1. No overall effect was detected, although there was a tendency towards reduced phosphorylation in scWAT of males was observed (Supplementary Fig. S2, Fig. 4g), which would be consistent with decreased activity of lipases and altered lipolysis. Consistently, no effect was observed in female samples, indicating differences in the effects of LPHN1 between sexes.

### A genetic *LPHN1* variant identified in an individual suffering from childhood overweight shows impaired expression and signaling

**Fig. 5:**
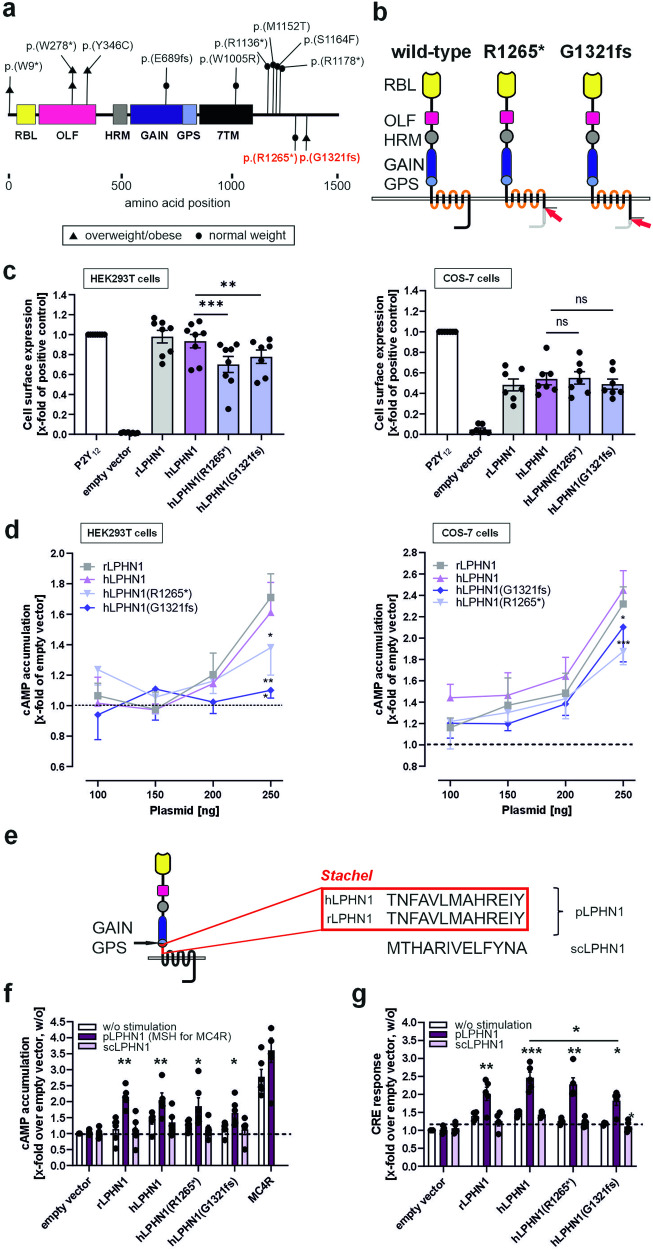
Variants of human *LPHN1* discovered in cohort analysis show altered expression and signal transduction. **a** Different *LPHN1* variants were previously linked to neurodevelopmental defects^52^ and are shown in black. Some of the patients also displayed overweight/obesity. Two novel variants identified in this study are located in the C terminus of the receptor and are shown in red. **b** Schematic depiction of the proteins resulting from the two *LPHN1* variants identified in children of the Leipzig Childhood Adipose Tissue cohort. The identified two variants contain a point mutation yielding a premature stop codon (R1265*) and a base insertion causing a frame shift that leads to a premature stop codon at amino acid position 1324 (G1321fs), respectively. The child carrying the latter variant is suffering from overweight/obesity. Both variants are located in the C terminus of the receptor (red arrows), thereby truncating it. Wild-type full-length human LPHN1 contains various domains: RBL, rhamnose-binding domain; OLF, olfactomedin domain; HRM, hormone-binding domain; GAIN, GPCR-autoproteolysis-inducing domain; GPS, GPCR proteolytic site. **c** Cell surface expression of rLPHN1, hLPHN1 and its two variants in HEK293T and COS-7 cells determined by ELISA. All receptors are detectable on the cell surface in both cell lines. While in COS-7 cells, no difference between the variants and the full-length wild-type receptor are present, in HEK293T cells, cell surface levels of the two variants are reduced. Data are displayed as percentage of the ADP receptor P2Y_12_ (positive control) and given as means ± SEM of at least six independent experiments, each performed in triplicate. The non-specific OD values (empty vector) are 0.001 ± 0.003 (HEK293T) and 0.025 ± 0.020 (COS-7) and the OD values of P2Y_12_ are 0.75 ± 0.07 (HEK293T) and 0.70 ± 0.11 (COS-7) (set 100%). ns = not significant; **p < 0.01; ***p < 0.001. **d** To test for functional coupling of hLPHN1 and the variants, accumulation of the second messenger cAMP was determined as a measure for G_s_ activation. The rat LPHN1 homolog rLPHN1^54^ served as positive control. Basal activity of hLPHN1 is even higher than rLPHN1 signals (in COS-7 cells), while LPHN1(R1265*) and LPHN1(G1321fs) signals are reduced in both, COS-7 and HEK293T cells. cAMP levels are displayed as means ± SEM of independent experiments, each performed in triplicate. Basal second messenger levels (250 ng empty vector) are 9.2 ± 6.3 nM cAMP (HEK293T) and 7.5 ± 5.9 nM cAMP (COS-7), *p < 0.05; ***p < 0.001, compared to the corresponding value of hLPHN1. **e** Schematic depiction of hLPHN1 with its tethered agonist (*Stachel*) sequence. This sequence, which also the sequence of the receptor-activating peptide pLPHN1, is located C-terminally of the cleavage site within the GPS and is identical to the one of rat LPHN1. scLPHN1 is a scrambled version of it. **f** The LPHN1 variants are activated by the *Stachel* sequence-derived peptide. The respective scrambled peptide of the same amino acid length and composition as the agonistic peptide served as negative control. HEK293T cells transfected with empty control vector (pcDps) or plasmid encoding the respective *LPHN1* variant were incubated with 1 mM of different peptides, and subsequently, cAMP concentrations were quantified as a measure for Gs coupling. As a positive control, the mouse melanocortin receptor 4 (MC4R) was used, which was incubated with 10 µM α-MSH. The basal cAMP levels (empty vector, no peptide) are 94.7 ± 12.9 nM. Data are given as means ± SEM of four independent experiments, each performed in triplicate. *p < 0.05; **p < 0.01 compared to the respective unstimulated control. **g** CRE reporter gene assays reveal different activation of LPHN1 variants. HEK293T cells transfected with empty vector control (pcDps) or plasmid encoding the respective *LPHN1* variant together with a vector carrying a CRE-luciferase were incubated with 1 mM of LPHN1-activating or scrambled peptide, respectively. Subsequently, luciferase activity was measured. Data is presented as fold changes over empty vector control without stimulation and given as means ± SEM of four independent experiments, each performed in triplicate. *p < 0.05; **p < 0.01: ***p < 0.001 compared to the respective unstimulated control if not indicated otherwise. Basal signals of the reporter assay (empty vector, no peptide) are 6,415 ± 182

The findings regarding body weight and alterations in metabolism in LPHN1-deficient mice prompted us to screen human cohorts for body weight effects associated with *LPHN1* variants. In a cohort of 81 children, we identified two truncating variants in *LPHN1*: NM_001008701.3:c.3976 G > GC, p.Gly1321*fs* and NM_001008701.3:c.3808 C > T, p.Arg1265*. Both truncating mutations led to a premature stop of translation in the C terminus of the receptor protein downstream the seven-transmembrane helix domain (Fig. 5a). Variant p.Gly1321fs was identified in a female (age: 13.8 years) suffering from overweight (BMI-SDS: 1.672), variant p.Arg1265* was identified in a younger female (age: 7.2 years) with normal weight (BMI-SDS: 0.807). As no access to other members of the families was available to segregate the two variants, we could not clarify whether the variants were inherited or de novo. However, the variants were not found in the general population. The *Lphn1* gene has a Z-score of 3.43 and a pLi of 1,^51^ suggesting that variants and loss-of-function mutations are not well tolerated. Indeed, a recent study showed that haploinsufficiency caused by several mutations (Fig. 5a) leads to neurodevelopmental delays in humans.^52^ The variants we identified are located more C-terminally to the ones described in the aforementioned study (Fig. 5a, b). This likely leads to a partial loss-of-function of the full-length receptor, or might allow for splice variants containing only the N-terminal regions of the receptor to remain functional. The existence of such variants has previously been shown.^53^ Both scenarios can contribute to a less severe or no neurodevelopmental phenotype in individuals carrying the mutants we identified.

As the truncated variants were identified in children with different weight phenotypes, we questioned whether and to which extent the *LPHN1* variants display impaired functionality. To address this question, we studied their signaling properties. First, we generated the human full-length wild-type (Ensembl ID ENST00000361434.8) and mutant (hLPHN1(R1265*), *hLPHN1(G1321fs)*) receptors. All three constructs were expressed in COS-7 and HEK293T cells, and cell surface expression was assessed. While wild-type full-length hLPHN1 was present at similar levels as previously described for the rat homolog rLPHN1,^54^ the two variants led to a reduced expression in HEK293T cells (Fig. 5c). Interestingly, this difference was not observed in COS-7 cells (Fig. 5c), most probably because of cellular saturation of protein synthesis and transport to the plasma membrane or due to a different repertoire of cellular components in these cells compared to HEK293T cells. Next, we tested for G-protein coupling by measuring basal activity. Basal activity refers to the activity of a GPCR in the absence of an agonist. Thereby, the GPCRs exist in an equilibrium of inactive and active conformation with few receptor molecules residing in the active state.^55^ Overexpression of the receptor increases the number of molecules in both states proportionally, and, at a certain level, the signal elicited by the receptors in the active state can be detected. To measure basal activity of hLPHN1, hLPHN1(R1265*), and hLPHN1(G1321fs), we determined the level of second messenger cAMP, which is downstream of the Gs-protein pathway. As the hLPHN1 amino acid sequence (product of Ensembl ID ENST00000361434.8) is more than 98% identical to the rLPHN1 (product of Ensembl ID ENSRNOT00000103294.1), it is highly likely that both signal via the same G-protein pathway. For rLPHN1, we previously showed that the receptor activates G_s_-protein cascades.^54^ Thus, we determined basal activity of hLPHN1 by measuring the levels of the second messenger cAMP, which increase upon activation of the G_s_-protein pathway. We found evidence that hLPHN1 indeed coupled to G_s_ (Fig. 5d). Interestingly, in HEK293T cells we observed for hLPHN1(R1265*) a reduced ability to activate the G_s_ pathway while no activation was observed for hLPHN1(G1321fs) (Fig. 5d). This reduction in signal might be partially due to the reduced expression observed in HEK293T cells (Fig. 5c). However, given the comparable expression of both variants in these cells, hLPHN1(G1321fs) appeared to have an impaired function independently of the expression level (Fig. 5d).

It is well-established that latrophilins are activated by a sequence located within the GPCR proteolytic site (GPS), an integral motif of the GPCR autoproteolysis-inducing (GAIN) domain (Fig. 5e).^54,56^ Synthetic peptides derived from this so-called *Stachel* sequence are able to activate the receptor even in the absence of other ligands.^54,56,57^ As the potential *Stachel* sequence of hLPHN1 is 100% identical to the one of rLPHN1 (Fig. 5e), it is likely that not only the signaling cascade mediated by the two homologs is identical, but also the activation mechanism. To test whether this sequence is able to activate hLPHN1 as well as the two identified variants, we treated them with the 13-amino acid-long *Stachel* sequence-derived peptide (referred to as pLPHN1) or a scrambled version (scLPHN1) (Fig. 5e) and measured cAMP accumulation. The wild-type hLPHN1 was activated by the peptide to a similar extent as the rat homolog (Fig. 5f). Likewise, both identified variants retain the capability of being activated by this sequence. A reporter gene assay measuring the downstream effects of the G_s_ signal by detecting CRE-mediated luciferase expression confirmed that especially hLPHN1(G1321fs) displays reduced functionality (Fig. 5g).

It should be noted that a recent publication claims that LPHN1 reduces levels of cAMP when activated by glucose.^58^ We, therefore, tested this in our system and measured cAMP accumulation in *LPHN1*-transfected HEK293T cells upon glucose stimulation, however, we did not observe significant changes in cAMP levels (supplementary Fig. S3a, b). To exclude that the chosen cell-type is responsible for the missing stimulation, we repeated the experiment using transfected CHO cells. Here, we also observed that glucose indeed reduced cAMP in CHO cells co-stimulated with forskolin, however, this effect was not specific to cells transfected with *LPHN1* (supplementary Fig. S3c). Furthermore, we did not observe any increase in cAMP due to glucose stimulation (supplementary Fig. S3d). Additionally, we also verified receptor cell surface expression after transient transfection (supplementary Fig. S3e). Taken together, our findings do not confirm that LPHN1 is a glucose-sensing GPCR.

These data show that hLPHN1 is activated by a *Stachel* sequence-derived peptide and signals via the G_s_-protein pathway similar to its previously described rat homolog.^54^ While the two variants in hLPHN1 yield receptors displaying the same general signaling route, hLPHN1(G1321fs) expression and function in HEK293T cells were substantially reduced. Together with our observation that the complete absence of LPHN1 resulted in increased age-dependent weight gain in mice, loss-of function of hLPHN1 may contribute to the development of obesity in humans.

## Discussion

The body’s energy balance, and thus, stable weight, strongly depends on a multitude of processes. One of the most prominent ones is food intake. The underlying mechanisms are tightly controlled by central and peripheral signaling pathways. These include direct hypothalamic cascades such as the leptin/ghrelin and the NPY system, or activation and inhibition of the MC4R. It further also entails metabolic processes such as lipolysis. Here, we identified LPHN1 as a novel player in the modulation of food intake and energy balance.

*Lphn1* knockout mice become severely overweight with age, a condition paralleled by increased food intake, leading to a disturbed energy balance and obesity accompanied by general features of this disease, such as altered hormone levels (Figs. 1, 2, 4). This set of phenotypes is comparable to the one observed in other obese mouse models like *ob/ob* mice that have an impaired leptin production,^43,59^ insulin insensitivity, and impaired glucose tolerance. Thus, it was conceivable that LPHN1 controls food intake in a similar manner. The receptor, which belongs to the adhesion class of GPCRs, has been previously shown to be highly expressed in the brain,^60^ specifically in neurons.^38,39^ Although analysis of existing RNA-seq datasets^45^ revealed high expression in POMC- and AGRP/NPY neurons (Fig. 3), which are the main regulators of food intake,^61^ the regulation does not affect expression levels of hormones, such as POMC, AGRP, or NPY (Fig. 3) but instead seems to route via a different mechanism. Our data further demonstrate, that also the expression of downstream neuropeptides involved in the regulation of food intake/energy homeostasis is not altered in *Lphn1* knockout mice.

Besides the similarities of *Lphn1* knockout mice to other obese mouse models, there are also distinct differences in the phenotype, which led us to hypothesize that LPHN1 might fulfil more than a hypothalamic function in the modulation of food intake. This is further strengthened by a recent study of a hypothalamic *Lphn1* knockout mouse model where female mice are protected from obesity.^58^ Our analysis reveals that Lphn1 knockout mice exhibit higher carbohydrate oxidation compared to fat oxidation (Fig. 2), in contrast to *db/db* mice, which carry a mutation in the leptin receptor and demonstrate the opposite metabolic pattern.^62^ Generally, it was anticipated that mice would metabolize more fatty acids than carbohydrates due to the presence of accumulated fat. Given that mice lacking LPHN1 also exhibit lower serum levels of non-esterified fatty acids (Fig. 4), it is plausible that the receptor could influence lipid metabolism or storage, thereby modulating food intake. Studies show that FFA receptors FFAR1 and FFAR4 are expressed in the hypothalamus and their activation reduces food intake.^22^ This effect has also been known to exist in mice lacking the adipose triglyceride lipase (ATGL).^63^ These mice have a drastically reduced capability to hydrolyze triacyl glycerides resulting in accumulation of fat, increased food consumption, and, consequently, overweight.^64,65^ As the absence of LPHN1 leads to reduced lipase levels (Fig. 4) and the receptor is expressed in adipocytes,^37^ it is conceivable that it might regulate lipolysis. Besides the expression regulation of lipolytic enzyme, the loss of direct impact on lipolysis via Gs protein-mediated cAMP increase would also be an explanation. Nevertheless, it’s important to consider that the increased insulin levels in *Lphn1* knockout mice may contribute to the observed reduction in fatty acid usage, as insulin is known to be one of the strongest inhibitors of lipolysis. As direct regulatory effects on lipid (and peripheral) metabolism exerted by the hypothalamus have been reported,^66,67^ a direct influence of LPHN1 from hypothalamic neurons might also be possible. In this context, it is noteworthy that a recent study describes LPHN1 to be a glucose sensor in neurons of the brain.^58^ However, we were not able to provide supporting experimental evidence for such LPHN1 function (supplementary Fig. S3). Albeit no gross phenotypic differences between male and female *Lphn1* knockout mice were observed (Figs. 1, 2), interestingly, the effects on lipid metabolism seem only to be pronounced in male mice, not so much in females, suggesting a sex-specific mechanism. However, since estrogen has been shown to already decrease basal lipolysis, this might complicate the detection of the differences.^68^ Previously, we also found that LPHN1 modulates insulin secretion from pancreatic β cells.^56^ It will be interesting to see whether there is an interplay between the regulation of energy and glucose homeostasis. Future comprehensive studies, employing approaches such as conditional knockout of *Lphn1* in the hypothalamus, adipose tissue, and pancreatic cells, will elucidate the contribution of different receptor functions.

Consistent with our findings on the role of LPHN1 in the regulation of energy balance and food intake in mice, the analysis of human adipose tissue RNA-seq data from the Leipzig Childhood Adipose Tissue cohort^69,70^ revealed two novel rare variants of *LPHN1* with one of these identified in a child suffering from overweight/obesity (hLPHN1(G1321fs)). Subsequent functional analysis of these two variants revealed that hLPHN1(G1321fs) completely lacks basal activity, while hLPHN1(R1265*) exhibits decreased functionality, accompanied with reduced surface expression in both cell lines. Although activation by the agonistic peptide is still possible, the effect is reduced, particularly in hLPHN1(G1321fs) (Fig. 5f, g). Especially the fact that in the monkey cell line COS-7, expression of the variants is not hampered, but signaling still is, indicates that in vivo (in humans) this reduced receptor functionality contributes to the observed phenotype.

Interestingly, the child suffering from overweight/obesity is older, which is similar to Lphn1 knockout mice that do not become overweight at very young age, but only around 7–12 weeks. Thus, it can be speculated that the child carrying hLPHN1(R1265*) may still develop obesity. This suggests that mutations within this aGPCR’s coding sequence might constitute a risk factor for developing obesity, and it is conceivable that its role in humans is akin to that observed in mice.

In line with the identification of an obesity-related *LPHN1* variant, the locus of *LPHN1* (19p13.3-p13.11) has already previously been linked to childhood obesity.^71^ Furthermore, in a study on the effects of LPHN1 on cognitive function that characterized ten human individuals with different *LPHN1* mutations causing neurodevelopmental disorders, four out of these patients were reported to be overweight.^52^ Thus, it seems likely that the mutations in LPHN1 might contribute to the overweight development, although it was not investigated in the respective study. Likewise, there is no information on the cognitive abilities of the patients in the Leipzig Childhood Adipose Tissue cohort nor on the parents. Hence, a potential link between the function of LPHN1 in neurodevelopment, cognitive function, and food intake can be postulated, but further analyses are warranted. Additionally, the *LPHN1* mutations we identified in the present study are located further downstream in the C terminus of the receptor compared to the ones described to cause neurodevelopmental disorders (Fig. 5), potentially yielding smaller alterations of the receptor’s function or not impairing splice variants only comprising the N-terminal regions of the aGPCR. Nevertheless, it is worth noting that inactivating mutations within *LPHN1* and *LPHN1* pseudogenization are generally rare in humans and vertebrates,^72,73^ respectively. This might explain the missing association of *LPHN1* variants with obesity and metabolic dysfunctions, e.g., in DisGeNET, a database for gene/variant associations with human diseases.^74^ In vitro analysis of the two rare variants show that residual receptor activity is retained in both variants, which is in line that complete loss-of-function mutations is not well compatible with healthy life.

In the case of the two LPHN1 variants identified here, we observed a reduced expression compared to the wild-type receptor in both variants, however, basal signaling properties were almost completely lost hLPHN1(G1321fs) in the variant identified in child suffering overweight/obesity. The fact that only their C termini are shortened, but the rest of the receptor remains unaltered, explains why both human LPHN1 variants can still be activated by *Stachel* sequence-derived peptides.

In conclusion, while *LPHN1* variants are rare and only a few cases linking them to obesity exist thus far, when considered alongside our findings in *Lphn1* knockout mice, they underscore the significance of the receptor in the regulation of food intake and energy homeostasis.

Our data indicate that LPHN1 signaling modulates energy balance mainly through food intake. The observed obese phenotype in mice lacking LPHN1 function is potentially caused by this imbalance and it can be speculated that this might also be the case in humans. Thus, it can be hypothesized that the human *Lphn1* variants are contributing to the onset and manifestation of obesity which might progress with age. It is not reported whether these children were suffering from other conditions that might be related to the receptor malfunctioning. However, as the individuals were only heterozygous for the mutated variant, the wild-type full-length copy might be sufficient to fully maintain other receptor functions. Furthermore, numerous different transcript variants have been reported for *Lphn1*.^53^ Thus, it is conceivable that in different cellular and functional contexts, distinct receptor variants maybe differently affected by the potentially truncating mutations. Further studies need to concentrate on LPHN1’s detailed function including its activation mechanism.

## Materials and methods

### Materials

Standard materials were purchased from Merck or Carl Roth. Primers were designed using NCBI PrimerBlast and synthesized by Microsynth.

### Animals

B6;129S6-*Adgrl1*^tm1Sud^/J mice (JAX stock number #006393) were obtained from the Jackson laboratories. Mice were bred in a specific pathogen-free environment under a 12:12 h light/dark cycle at 21 °C and 55% humidity. They had *ad libitum* access to water and food (standard diet “rat/mouse maintenance feed”, SSNIFF: 17.1 MJ/kg gross energy, 23.0% protein, 6.1% fat, 5.1% sugar). Wild-type (WT) and knockout (KO) siblings used in this study were obtained by breeding heterozygous animals. Individuals were genotyped with the primers and a protocol provided by JAX (Protocol 22565: Standard PCR Assay - Adgrl1<tm1Sud> Version 1.3). For all experiments, 30–34-week-old mice were used unless stated otherwise. Breeding, experimental procedures and sacrificing were performed in accordance with European Directive 2010/63/EU on the protection of animals used for scientific purposes and were conducted with permission from the local Animal Care and Use Committee (ACUC T19/18; TVV43/18; TVV28/19; TVV26/22) and the Government of the State of Saxony, Germany.

### Determination of body and organ weight, length, and body composition

Mice were weighed weekly after weaning starting at 3 weeks of age. At the age of 30–34 weeks, whole body fat composition was measured individually using the EchoMRI body composition analyzer system (Echo Medical Systems). Subsequently, mice were sacrificed and body length was measured from nose-tip to tail-base. Tail length was measured from the tail-base to the tail-end. Pancreas, liver, left/right kidney, heart, spleen, and brain were removed and weighed. Parts of the liver were retrieved for compositional analysis with EchoMRI and histology.

### Metabolic characterization

Energy consumption, energy expenditure, substrate utilization (respiratory exchange ratio), oxygen consumption, and home-cage activity were measured in temporally single-housed mice using a climate-controlled indirect calorimetry system (TSE System, Bad Homburg, Germany). Analyses were carried out using ANOVA and the R-based CalR package with body weight as covariate.^75^ Whole-body fat and carbohydrate oxidation rates [g/min] were calculated using the equations 1.695xV_O2_-1.701xV_CO2_ and 4.585xV_CO2_-3.226xV_O2_ with V_O2_ and V_CO2_ given in l/min, respectively.^76^

### Glucose and insulin tolerance tests

For intraperitoneal glucose tolerance tests (ipGTT), mice were fasted for 16 h overnight. Subsequently, 5 µl of a 20% glucose solution (Glucosteril, Fresenius Kabi) per g body weight were injected intraperitoneally. Blood was withdrawn from the tail vein and glucose concentrations were measured before and 20, 40, 60, and 120 min after glucose application (ACCU-CHEK Performa, Roche).

For insulin tolerance tests (ITT), mice were fasted for 6 h before intraperitoneal injection of 1 mU human insulin/g body weight (Insuman Rapid, Sanofi). Blood glucose levels were measured from tail vein blood before and 15, 30, 45, and 60 min after insulin application (ACCU-CHEK Performa, Roche).

### Fasting insulin measurements

Fasting insulin content was measured in blood plasma in overnight fasted 30–34-week-old mice. Thereto, plasma samples were analyzed using the Ultra Sensitive Mouse Insulin ELISA Kit (Crystal Chem, #90080) according to the manufacturer’s instructions.

### Serum analyses

Blood was terminally collected from 30–34-week-old mice and serum was taken. Serum samples were analyzed in duplicates using magnetic bead-based multiplex immunoassays (Bio-Plex Pro Mouse Diabetes 8-Plex Assay, #171F7001M and Pro Mouse Diabetes Adiponectin Assay, #171F7002M, Biorad) according to the manufacturer’s instructions. Analyses were performed using the Bio-Plex200 suspension array system (Biorad). Analyte concentrations (pg/ml) were calculated from the appropriate optimized standard curves with the help of the Bio-Plex-Manager-Software version 6.0 (Biorad).

Non-esterified fatty acids (NEFA) were measured using the Wako HR NEFA Kit (Fujifilm) according to the manufacturers’ protocol. A standard series comprising 0, 0.125, 0.25, 0.5, and 1 mM NEFA Standard was prepared and 4 µl serum or standard and 200 µl Fujifilm NEFA (HR) 1 were incubated for 5 min at room temperature. Samples were measured using the Synergy microplate reader (BioTek) at 550 nm. Subsequently, 100 µl NEFA (HR) 2 was added and samples measured after another 5-minute incubation at room temperature.

### Oil red O staining of liver sections

Samples of the middle liver lobe of mice were transferred to blisters with Tissue-Tek (Sakura) and frozen in liquid nitrogen. Frozen tissue samples were cut into 10 μm-thick sections and mounted on glass slides. Before staining, the sections were air dried, fixed with 4% paraformaldehyde for 5 min, washed under running tap water for 10 min, and immersed in 60% isopropanol for 5 min. Staining was performed in freshly prepared 0.3% Oil Red O / 36% isopropanol in water solution for 15 min and followed by briefly rinsing with 60% isopropanol. Oil Red O-stained sections were transferred to distilled water for 10 min, counterstained with Mayer’s hemalum solution and embedded with glycerol gel. Sections were analyzed under an Axioplan 2 microscope (Zeiss) equipped with a ProgRes C3 digital camera (Jenoptik) connected to a digital recording system (ProgRes CapturePro 2.8.8; Jenoptik).

### RNA isolation, reverse transcription, and quantitative PCR (qPCR)

RNA was isolated from the hypothalamus and visceral adipose tissue (vWAT) of 30–34-week-old mice. Mice were sacrificed, the brain was removed, dissected, and both tissues immediately frozen in liquid nitrogen. RNA isolation from hypothalamus was performed using TRI-Reagent (Sigma-Aldrich) according to the manufacturer’s instructions. Adipose tissue RNA was isolated using the SV Total RNA Isolation System (Promega). RNA was transcribed into cDNA by reverse transcription using Superscript II Reverse Transcriptase (ThermoFisher) and a mixture of oligo (dT) and random hexamer primers according to the manufacturer’s protocol. mRNA expression of genes was determined by qPCR with specific primers (supplementary Table S1) for the respective genes using the Luna Universal qPCR Master Mix (New England BioLabs) according to manufacturer’s protocol and the CFX Connect Thermal Cycler (BioRad). For primer sequences, see supplementary Table S1. Data analysis was performed with the CFX Manager (Bio-Rad).

### Western blot analyses

Visceral (vWAT) and subcutaneous (scWAT) white adipose tissue was removed from 30–34-week-old mice and lysed using TissueRuptor II (Qiagen) in 500 µl lysis buffer (20 mM Tris-HCl, 1 mM EDTA, 255 mM sucrose, pH 7.4) containing Halt Protease and a Phosphatase Inhibitor Cocktail (ThermoFisher) to inhibit proteinases and phosphatases. Protein concentration was determined by Pierce BCA Protein Assay Kit (ThermoFisher), samples were diluted in 4x Laemmli buffer (250 mM Tris (pH 6.8), 8% SDS, 20% glycerol, 0.02% bromophenol blue, 100 mM DTT) and boiled (95 °C, 5 min). Proteins were separated by SDS-PAGE and semidry-blotted (Peqlab) on nitrocellulose membrane. Total protein on the membrane was stained using the Revert 700 Total Protein Stain and Wash Solution Kit (LI-COR) for normalization. Non-specific binding sites were blocked with Intercept Blocking Buffer (LI-COR) for 1 h. The blot was incubated with primary antibody (1:1000, Phospho-PKA Substrate (RRXS*/T*) (100G7E) rabbit mAb, Cell Signaling) overnight at 4 °C. After washing with TBST (3×10 min), the membrane was incubated with secondary antibody (1:2000, IRDye 800CW Goat anti rabbit, LI-COR) for 1 h at room temperature and washed with TBST (3×5 min). Imaging was performed with LI-COR Odyssey Imaging System 9120.

### Identification of genetic *LPHN1* variants found in children with obesity

Rare genetic variants were detected in human RNA-Seq samples obtained from subcutaneous adipose tissue of subjects of the Leipzig Childhood Adipose Tissue cohort (40 lean patients (18 female, 22 male) and 41 patients with overweight/obesity (21 female, 20 male); NCT02208141, 265/08-ek).^69,70^ In this cohort of 81 children, RNA-seq from fat tissue was performed as previously described.^77,78^ We mapped RNA-seq reads with STAR v2.7.9a using the twopassMode option and otherwise standard parameters.^79^ Transcriptomes showed a mean of 30× -coverage. We used gatk v4.2.0 for transcript variant calling,^80^ we excluded soft clipped bases and required a minimum confidence of 20 (PHRED scale) for variant calling. We used SnpEff v5.1 for variant annotation and identified all variants in *LPHN1*.^81^ Furthermore, we considered only truncating variants with a genotype quality ≥ 30 (PHRED scale) and absent from GnomAD.^51^

### Cloning of latrophilin variants

Constructs for functional analyses were generated by cloning the *LPHN1* variants into the mammalian expression vector pcDps (Okayama and Berg, 1983), in which a *Bsr*GI site had been introduced into the multiple cloning site by outward PCR using phosphorylated primers fmi1_106F/fmi1_107R. The template DNA was eliminated by digestion with *Dpn*I and the vector was ligated. Human *LPHN1* cDNA (Ensembl ID ENST00000361434.8) carrying a hemagglutinin (HA) epitope tag following the signal peptide (predicted with SignalP 6.0) at codon position 28 and a C-terminal FLAG epitope tag was synthesized and cloned into a pUC57 vector (GenScript). Additionally, 5’ and 3’ of the *LPHN1* coding sequence, the construct contained a *Bsr*GI restriction site. *LPHN1* was released by cutting the synthesized construct with *Bsr*GI followed by gel-purification. In parallel, pcDps was digested with *Bsr*GI, dephosphorylated and ligated with *LPHN1*.

Mutant variants were generated by site-directed mutagenesis of the wild-type *LPHN1* construct (see above). To obtain hLPHN1(R1265*), a G to A point mutation was introduced by outward PCR with phosphorylated primers lat1_2339F/lat1_2340R. Likewise, for hLPHN1(G1321fs), a G was introduced after the C by outward PCR with phosphorylated primers lat1_2341F/lat1_2342R.

All constructs were verified by sequencing. For primer sequences see supplementary Table S2.

### Cell culture

COS-7 and HEK293T cells (ATCC) were grown in DMEM supplemented with 10% FBS, 100 U/ml penicillin, and 100 µg/ml streptomycin at 37 °C in a humidified incubator with 5% CO_2_. CHO cells (ATCC) were cultured in DMEM/F12 with 10% FBS, 100 U/ml penicillin, and 100 µg/ml streptomycin under the same conditions.

### Cell surface expression analysis by ELISA

COS-7 and HEK293T cells were split into 48-well plates (1.2 × 10^5^ cells/well), whereby the plates for HEK293T cells had been coated with 0.0002% poly-L-lysine (Merck) in PBS for 30 min at 37 °C prior to use. 24 h later, cells were transfected with 500 ng receptor-encoding plasmid DNA/well using Lipofectamine2000 (ThermoFisher) according to manufacturer’s protocol. To determine surface expression of human *LPHN1* and its variants, an indirect enzyme-linked immunosorbent assays (ELISA) was employed making use of the HA tag each construct harbors N-terminally. Transiently transfected COS-7 or HEK293T cells were fixed 48 h post transfection with 4% formaldehyde for 20 min, washed and incubated with blocking solution (DMEM + 10% FBS) for 1 h at 37 °C. ELISA was performed using an anti-HA-peroxidase-conjugated antibody 1:1000 (Roche) as previously described.^82^

### cAMP accumulation assay

For cAMP assays, cells were split into 96-well plates (1.5 × 10^4^ cells/well), whereby the plates for the HEK293T cells had been coated with 0.0002% poly-L-lysine (Merck) in PBS for 30 min at 37 °C prior to use. Cells were transfected with Lipofectamine2000 (ThermoFisher) according to manufacturer’s protocol 24 h later. For basal activity measurements, between 100 ng and 250 ng of DNA per well were transfected and for peptide activation detection, 100 ng DNA per well. 48 h post transfection, cells were washed with serum- and phenol red-free DMEM containing 1 mM 3-isobutyl-methyl-xanthine (IBMX) for 5 min. IBMX inhibits phosphodiesterases and subsequently blocks the degradation of cAMP. To analyze the effects of agonistic peptides (Peptides and Elephants GmbH), transfected cells were treated with 1 mM peptide in DMEM with 1 mM IBMX for 1 h at 37 °C.

To stop the incubation, the media was removed and the cells were lysed with LI buffer (PerkinElmer Life Sciences) and frozen at −20 °C. To measure cAMP concentrations, samples were thawed and the AlphaScreen cAMP assay kit (PerkinElmer Life Sciences) was applied following the manufacturer’s instructions in 384-well white OptiPlate microplates (PerkinElmer Life Sciences) utilizing the Spark Cyto multimode plate reader (Tecan).

### Reporter gene assay

HEK293T cells were seeded into 6-well plates (9 × 10^5^ cells/well) that had been coated with poly-L-lysine. After 24 h, the cells were co-transfected with 1.1 µg receptor plasmid/well and 4.4 µg of the plasmid encoding the CRE-luciferase per well using Lipofectamine2000 (ThermoFisher) following the manufacturer’s protocol. 5 h post transfection media was removed and replaced with phenol red-free standard media. Approximately 24 h later, cells were detached by incubation with 300 µl trypsin-EDTA/well (PAN-Biotech) for 5 min and 10^4^ cells in DMEM were seeded into the wells of a 384-well plate. After 16 h, stimulation was performed with 1 mM of the respective peptide in 15 µl DMEM for 2.5 h at 37 °C. Measurement of luciferase activity was performed using ONE-Glo reagents (Promega) according to manufacturer´s protocol by adding 15 µl of ONE-Glo reagent per well to the cells (without removing the medium before) and after 15 min, luciferase activity was measured with a Spark Cyto multimode plate reader (Tecan).

### Statistical analysis

Statistical and graphical analyses were performed using Prism version 10.0 (GraphPad Software). When comparing two groups, statistical significance was analyzed using the two-tailed unpaired t-test corrected for multiple testing using the Holm-Šídák method when necessary. When comparing two groups at multiple time points, a 2-way ANOVA was applied unless stated otherwise. Details are given in the figure legends.

### Supplementary information

Supplementary Materials

## Data Availability

All data supporting the findings of this study are available in the main text and its supplementary information. Raw data and further information is available from the corresponding authors on request.
