# Supplementary material for: Dysfunction of the adhesion G protein-coupled receptor latrophilin 1 (ADGRL1/LPHN1) increases the risk of obesity
Source: Signal Transduct Target Ther. 2024 Apr 26;9:103. doi: 10.1038/s41392-024-01810-7 (PMC11045723; doi:10.1038/s41392-024-01810-7)
Supplement: Supplementary file 1 — Supplementary Materials [file 41392_2024_1810_MOESM1_ESM.docx]

Supplementary Materials for

Dysfunction of the adhesion G protein-coupled receptor ADGRL1/LPHN1 increases the risk of obesity

André Nguyen Dietzsch, Hadi Al-Hasani, Joachim Altschmied, Katharina Bottermann, Jana Brendler, Judith Haendeler, Susanne Horn, Isabell Kaczmarek, Antje Körner, Kerstin Krause, Kathrin Landgraf, Diana Le Duc, Laura Lehmann, Stefan Lehr, Stephanie Pick, Albert Ricken, Rene Schnorr, Angela Schulz, Martina Strnadová, Akhil Velluva, Heba Zabri, Torsten Schöneberg, Doreen Thor, Simone Prömel

Correspondence to: proemel@uni-duesseldorf.de

doreen.thor@medizin.uni-leipzig.de

**This PDF file includes:**

Materials and Methods

Figures S1 to S3

Tables S1 to S2

Materials and Methods

Stimulation of LPHN1 with glucose

HEK293T cells transfected with either hLPHN1, rLPHN1, or control plasmid were incubated in glucose-free DMEM (Sigma-Aldrich) for 1 h and afterwards stimulated with increasing concentration of glucose with and without forskolin to assess activation of Gi or Gs proteins, respectively.

CHO cells were cultured in DMEM/F12 media supplemented with 10 % FBS, 100 U/ml penicillin, and 100 µg/ml streptomycin at 37°C in a humidified incubator with 5 % CO_2_. One day prior to transfection, cells were seeded into 96-well plates (1.2x10^4^ cells/well) for cAMP determination and 48-well plates (2.0x10^5^ cells/well) to assess cell surface expression. Cells were transfected with Lipofectamine2000 using 75 ng or 150 ng of plasmid DNA (rLPHN1, hLPHN1, empty vector). To analyze cell surface expression, ELISA was performed as described for HEK293T and COS-7 cells. To analyze glucose stimulation, cells were equilibrated in glucose-free HBSS/HEPES (137.9 mM NaCl, 1.26 mM CaCl_2_, 0.49 mM MgCl_2_, 0.41 mM MgSO_4_, 0.53 mM KCl, 0.44 mM KH_2_PO_4_, 0.34 mM Na_2_HPO_4_, 20 mM HEPES). Afterwards, stimulation with increasing amounts of glucose in HBSS/HEPES was performed for further 60 min. To assess the Gi-protein activation, all solutions contained 1 µM forskolin.


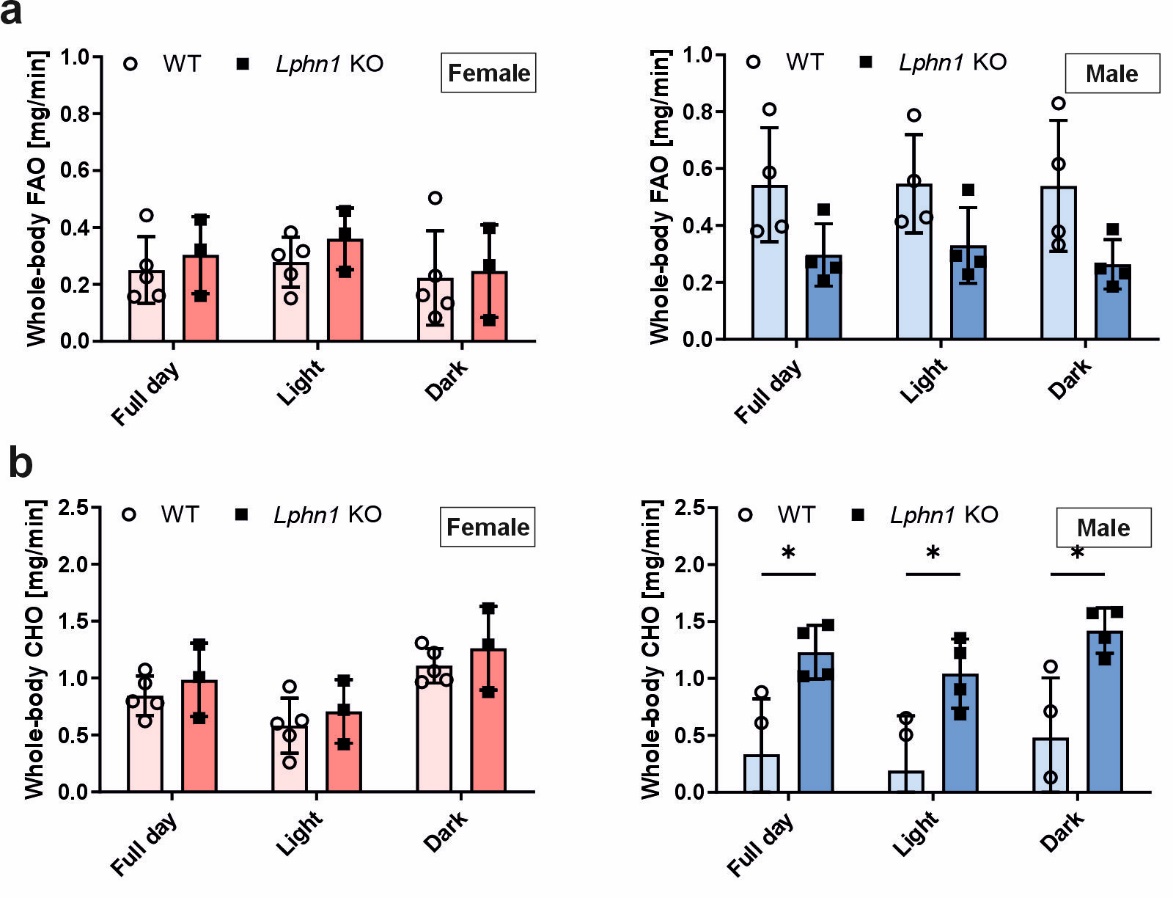


Figure S1. *Lphn1*-deficent mice display an increased weight and fat accumulation.

a) Whole-body fatty acid oxidation is reduced in male but not female *Lphn1* KO compared to WT siblings during light and dark phases. b) Simultaneously, whole-body carbohydrate oxidation is significantly higher in male but not female mice lacking LPHN1. Data are given as means ± SD of four 30 – 34-week-old males; * p < 0.05; ** p < 0.01; *** p < 0.001.


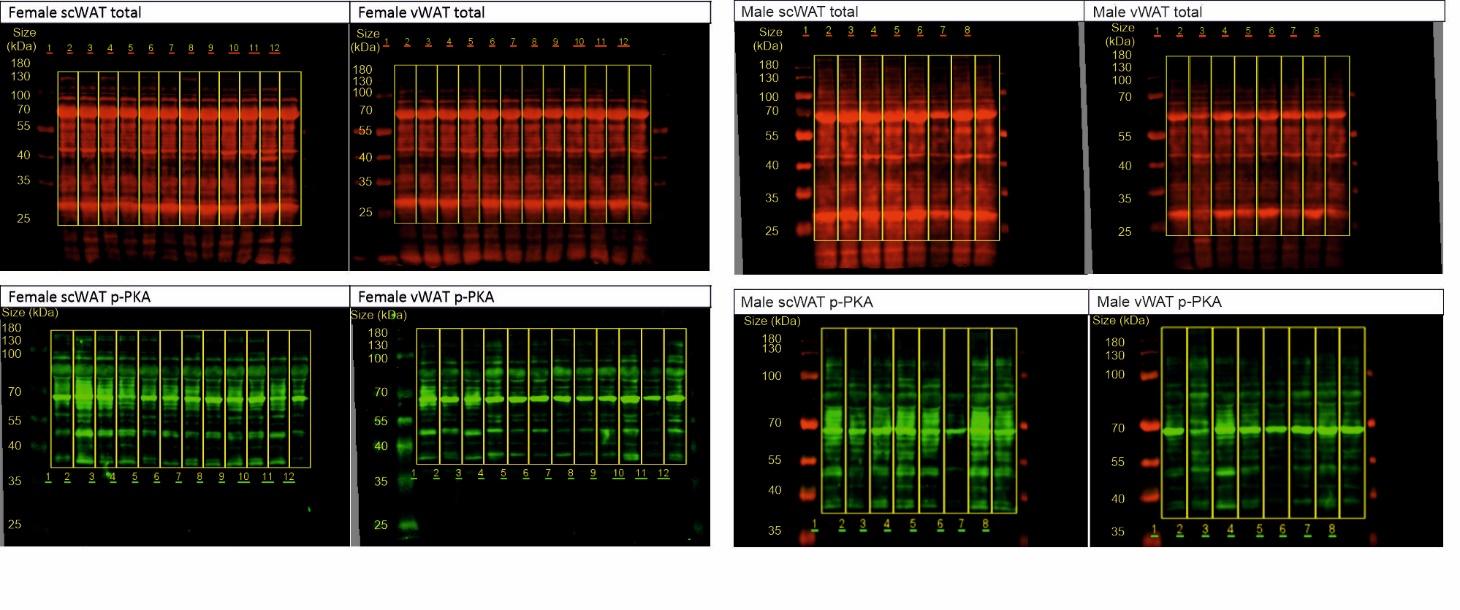


Figure S2. Western blots for the investigation of phospho-PKA substrates.

Proteins from visceral (vWAT) and subcutaneous (scWAT) white adipose tissue of female (left) and male (right) mice were blotted. Total protein on the membrane (red, top) stained as well as phospho-PKA substrates were visualized using an antibody specific for phospho-PKA substrates (green, bottom).


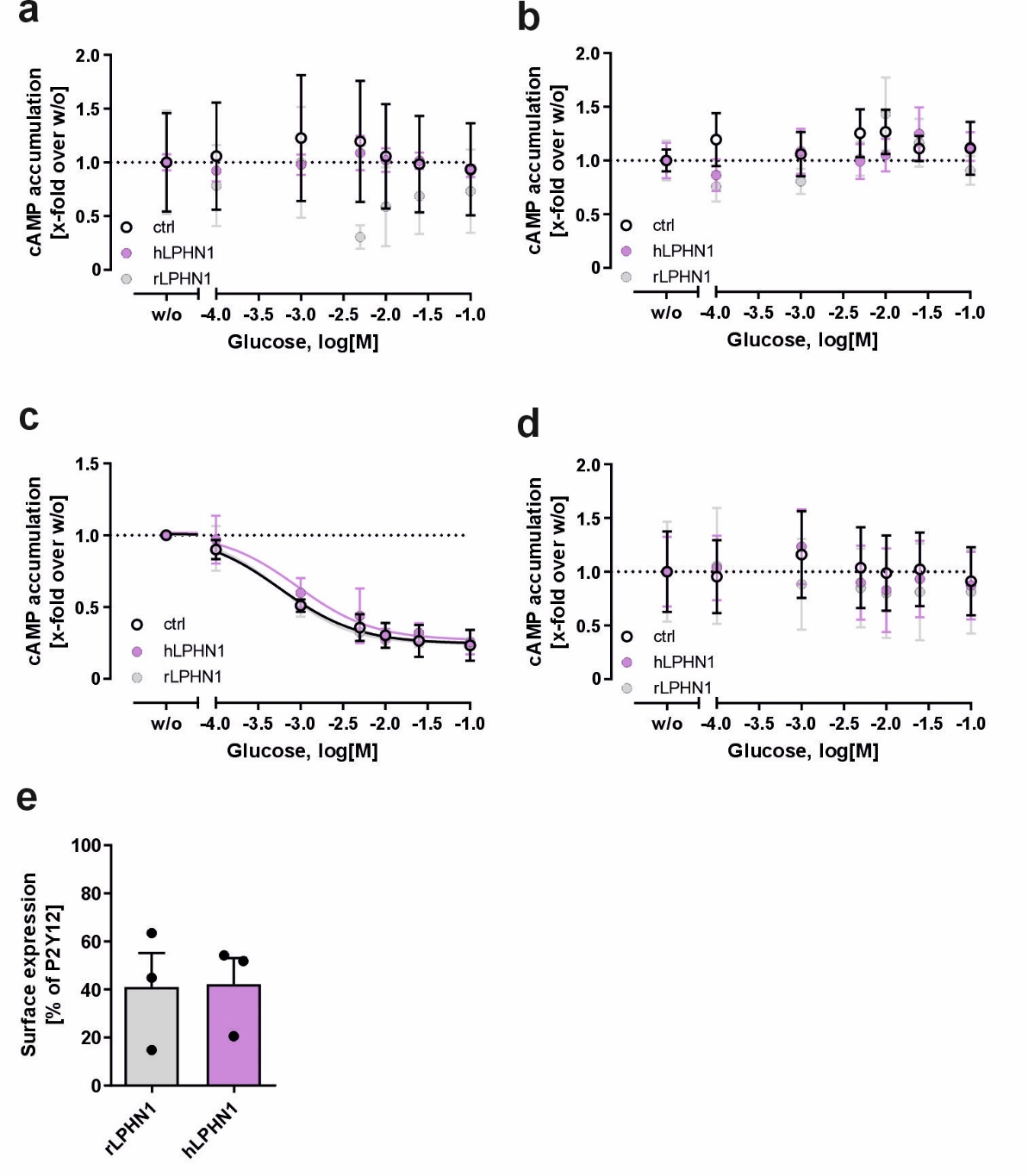


Figure S3. Glucose-induced changes in intracellular cAMP levels are not dependent on LPHN1.

a) and b) To assess glucose responsiveness of LPHN1 we stimulated transfected HEK293T cells with increasing amounts of glucose with (a) and without (b) forskolin and did not detect differences between control- and receptor-transfected cells. c) To assess Gi-protein coupling upon glucose stimulation, LPHN1- and control plasmid-transfected CHO cells were pre-incubated in glucose-free HBSS/HEPES for 60 min and afterwards stimulated with 1 µM forskolin and increasing amounts of glucose. d) To assess Gs-protein coupling upon incubation with glucose, LPHN1- and plasmid-transfected CHO cells were pre-incubated in glucose-free HBSS/HEPES for 60 min and afterwards stimulated increasing amounts of glucose. e) Cell surface receptor expression was determined in CHO cells demonstrating receptor expression. Given is the mean ± SEM of three independent experiments each performed in triplicates.

Table S1. Primer sequences used for qPCR analyses. Primer efficiency was tested prior to usage and only those having values ranging from 90% to 110% were considered for analysis.

| **Gene** | **Sequence forward primer (5’ - 3’)** | **Sequence reverse primer (5’ - 3’)** |
| --- | --- | --- |
| mouse *Actb* | GCTCTTTTCCAGCCTTCCTT | CGGATGTCAACGTCACACTT |
| mouse *Agrp* | AGACAACTGCAGACCGAGCA | CTTACACAGCGACGCGGAGA |
| mouse *Atgl* | CCACTGTCTTGCGCCACCTA | TGACGCTGGCATTCTTCCCA |
| mouse *B2m* | GCTATCCAGAAAACCCCTCAAA | GGCGGGTGGAACTGTGTTA |
| mouse *Cck* | GGTATTCGTAGTCCTCGGCACT | TAGCGCGATACATCCAGCAGGT |
| mouse *Hcrt* | AGCTGCGTGGTTACCGTTGGC | TGCCGTCTCTACGAACTGTTGC |
| mouse *Hsl* | CATCAACCGACCAGGAGTGCT | GCAGCCTTTGTGTAGCGTGA |
| mouse *Lpl* | ACAAGGTCAGAGCCAAGAGAAG | TGTTGCTTGCCATCCTCAGT |
| mouse *Mgl* | GCCCTCATCTTTGTGTCCCAT | GCAAATACCAGCATGTCCAGCC |
| mouse *Npy* | GGCATTCTGGCTGAGGGGTA | TGTAGTGTCGCAGAGCGGAG |
| mouse *Nts* | TGAGGCTGCAAACATTTAGCAAGG | AGAAGATGTGAGAGCCCTGGAG |
| mouse *Pcsk1* | ATCGCCGAAGAACTGGGGTATG | CTCCGAGGATGGCTTTTGTGTTT |
| mouse *Pcsk2* | TTGGCTACGGAGTCCTTGATGC | CTTGCCGGTGGGTGGTATTTT |
| mouse *Plin4* | TGTGACCAGCAGTGAAGATGC | GCTCCCATCACACCTCCAGAT |
| mouse *Pmch* | GGTTTTACAGCCAGACTCAGTGG | CTCTGGAACAATACAAAAACGACG |
| mouse *Pomc* | GACCTCACCACGGAGAGCAA | CTCCGTTGCCAGGAAACACG |

Table S2. Sequence of primers used to generate constructs presented in the study.

| **Primer** | **Sequence (5’ - 3’)** |
| --- | --- |
| fmi1_106F | GCTGTGTACACTGCAGCCCGGGAGATCTGG |
| fmi1_107R | GCAGTGTACACAGCTTTTAGAGCAGAAGTAACACTTCCGT |
| lat1_2339F | TGAGGCCGGAACCTAGCCGAT |
| lat1_2340R | GGGCGGCTCAGGGCCCCC |
| lat1_2341F | CGGGGTGCTGACCGGGCCGAG |
| lat1_2342R | CGGGCCCGCCCGCCTCTTCCT |
